# Perspectives on the Changing Landscape of Epizootic Hemorrhagic Disease Virus Control

**DOI:** 10.3390/v13112268

**Published:** 2021-11-12

**Authors:** Leela E. Noronha, Lee W. Cohnstaedt, Juergen A. Richt, William C. Wilson

**Affiliations:** 1 United States Department of Agriculture, Agricultural Research Service, National Bio and Agro-Defense Facility, Foreign Arthropod-Borne Animal Diseases Research Unit, Manhattan, KS 66502, USA; Leela.noronha@usda.gov (L.E.N.); Lee.Cohnstaedt@usda.gov (L.W.C.); 2 Center of Excellence for Emerging and Zoonotic Animal Diseases, and the Department of Diagnostic Medicine/Pathobiology, College of Veterinary Medicine, Kansas State University, Manhattan, KS 66506, USA

**Keywords:** white-tailed deer, cattle, epizootic hemorrhagic disease virus, EHDV, midges, *Culicoides*

## Abstract

Epizootic hemorrhagic disease (EHD) is an insect-transmitted viral disease of wild and domestic ruminants. It was first described following a 1955 epizootic in North American white-tailed deer (*Odocoileus virginianus*), a species which is highly susceptible to the causative agent of EHD, epizootic hemorrhagic disease virus (EHDV). EHDV has been detected globally across tropical and temperate regions, largely corresponding to the presence of *Culicoides* spp. biting midges which transmit the virus between ruminant hosts. It regularly causes high morbidity and mortality in wild and captive deer populations in endemic areas during epizootics. Although cattle historically have been less susceptible to EHDV, reports of clinical disease in cattle have increased in the past two decades. There is a pressing need to identify new methods to prevent and mitigate outbreaks and reduce the considerable impacts of EHDV on livestock and wildlife. This review discusses recent research advancements towards the control of EHDV, including the development of new investigative tools and progress in basic and applied research focused on virus detection, disease mitigation, and vector control. The potential impacts and implications of these advancements on EHD management are also discussed.

## 1. Introduction

Epizootic hemorrhagic disease virus (EHDV) belongs to the genus *Orbivirus* of the family *Reoviridae*. It is the causative agent of epizootic hemorrhagic disease (EHD), a disease of wild and domestic ruminants transmitted by *Culicoides* spp. biting midges (Diptera: Ceratopogonidae; [1]). North American white-tailed deer (WTD; *Odocoileus virginianus*) represent the species most severely impacted by EHDV, but several ruminant species, including domestic cattle, can also be infected and develop clinical disease (reviewed in [2]). EHDV infections in WTD can vary dramatically in presentation and range from asymptomatic or mild disease to severe hemorrhagic disease and acute death. In general, EHDV-infected cattle experience less severe disease, with some exceptions [3,4,5,6], including infections with Ibaraki virus (IBAV), a strain endemic to Asia which sporadically causes an acute febrile syndrome resembling bluetongue in cattle [7,8].

**Figure 1:**
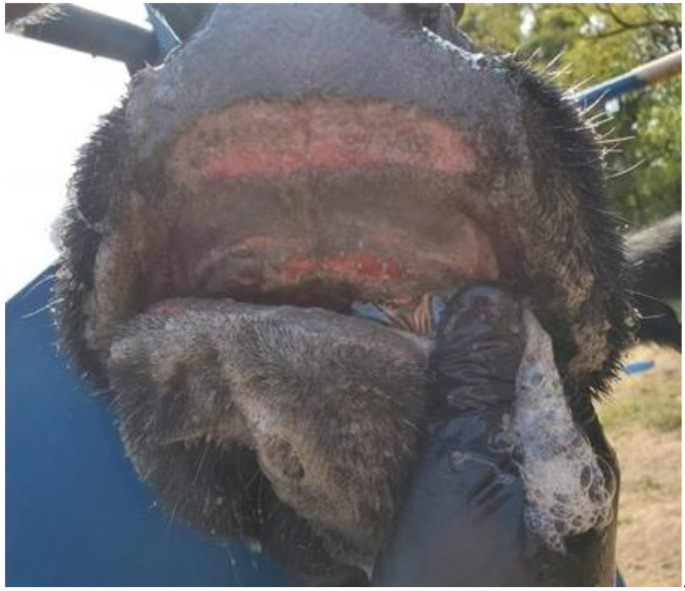
EHDV-induced oral lesions in cattle. Oral erosions and ulcerations observed in an Angus cow during a 2019 EHDV outbreak in Harrison County, West Virginia (WV), USA. This cow also exhibited lameness, drooling, respiratory distress, fever, and anorexia. The case symptomatology initiated a foreign animal disease investigation by the WV Department of Agriculture Animal Health Division and the U.S. Department of Agriculture Animal and Plant Health Inspection Service. EHDV was confirmed on this farm and two other Harrison County farms. The WV Division of Natural Resources also confirmed EHDV in WTD following deaths in wild deer populations in multiple regions across the state, including the areas of the affected cattle farms. EHDV serotype 2 viruses were detected in WTD samples and one cattle sample. Photo: Dr. Robert Stenger (used with permission).

EHDV was first identified following a 1955 United States (U.S.) outbreak in WTD, with deer mortality so remarkable that it was initially investigated as a mass intoxication event [9,10]. Descriptions of disease consistent with EHD, however, date back to at least 1890 [10]. Since that time, EHDV has become widespread across the U.S. and has had a growing global presence. Despite EHDV’s extended presence in the U.S., methods to prevent and treat EHD are still limited; there is a pressing need for new approaches to mitigate EHDV epizootics, which can be devastating for animals and stakeholders. As evidenced by the first documented outbreak, the impacts of EHDV in susceptible WTD populations can be severe, with outbreak-related mortality estimates of up to 20% in wild deer [11]. EHD is also the most severe disease of captive deer, where sporadic epizootics can cause significant economic losses to deer farming operations [12,13]. Although EHD in cattle is typically milder, it can produce oral lesions that resemble transboundary vesicular diseases such as foot-and-mouth disease (Figure 1), leading to foreign animal disease investigations of affected premises that are costly and restrict the movement of livestock [14]. Additionally, concerns about potential increases in EHDV virulence in cattle have been raised in the last two decades following reports of clinical disease during recent outbreaks in North America, the Mediterranean Basin, and Reunion Island [3,15,16,17]. Inactivated and live-attenuated (modified live) virus vaccines are available for control of IBAV in cattle in Japan [18]. In North America, the absence of licensed commercial EHDV vaccines has historically focused control methods on the management of insect vectors.

In 2015, the state of the EHDV research field was explored through several comprehensive reviews as well as the published outcomes of a 2013 gap analysis; topics included vector management, diagnostic tools, vaccines, transmission, and other knowledge gaps [15,17,19,20,21,22,23]. Since then, EHDV research has been highly active with new developments in most major areas. Several new tools and models have been developed and used to generate discoveries with the potential to translate into applied methods to control EHDV transmission and clinical disease. This review discusses some of the research developments from 2016 to present that may inform efforts toward the management and control of EHD.

## 2. EHD’s Evolving Epidemiology

The changing patterns and distribution of EHDV provide relevant context in approaching effective virus control strategies, as well as highlight the inherent challenges that these strategies face (Table 1). Seven serotypes of EHDV, numbered 1, 2, and 4-8, are currently recognized [24]. An additional serotype designation was previously proposed by Campbell and St. George [25]; however, genetic analyses later showed the proposed EHDV serotype 3 to be a serotype 1 virus. Investigations of a 1955 outbreak of hemorrhagic disease in North American wild ungulates led to the first isolation of EHDV, later classified as EHDV-1 [10]. An outbreak in 1962 in the Canadian Province of Alberta resulted in the isolation of a second serotype of the virus, designated EHDV-2 [26,27]. Historically, EHDV-1 and -2 were the only serotypes known to circulate in North America, until serotype 6 EHDV was first detected in 2006 in the Midwest. The EHDV-6 serotype virus, which was determined to be a reassortant of EHDV-2 with an exotic EHDV-6, has since become established across the U.S. [28,29]. EHDV has been known to circulate outside of North America since shortly after its initial discovery. A 1959 outbreak of acute febrile illness in Japanese cattle led to the identification of IBAV, which was subsequently shown to be antigenically related to, and now reclassified as, EHDV-2 [30,31,32]. Globally, EHDV has been identified broadly across temperate and tropical regions of the Americas, Asia, Africa, Australia, and the Middle East (detailed in [33]). Recent studies seem to have confirmed emerging concerns regarding shifts in historical patterns of EHDV distribution and epidemiology. Detection of EHDV-2 genomes and antibodies in WTD and cattle in east-central Canada suggest a potential progression of the previously observed northern expansion of virus occurrence in North America [34,35,36,37]. Globally, evidence of EHDV circulation was recently documented for the first time in Ecuador (EHDV-1), Zimbabwe (serotype not determined), the island of Mayotte (EHDV-6), Trinidad (EHDV-6), French Guiana (EHDV-1 and -6) and Egypt (EHDV-1), all in samples (some from historical collections) taken from cattle [38,39,40,41,42,43,44]. The introduction of new serotypes has recently been detected in Israel (EHDV-1 and -6) and China (EHDV-7), also identified in cattle samples [45,46,47]. It has been suggested that some EHDV isolates collected from cattle in Japan and China may also represent novel serotypes based on genomic analyses and a lack of cross-neutralization with known serotypes [48,49]. The majority of the cattle from which the EHDV-positive samples were obtained reportedly were asymptomatic. A notable exception to this was Israel, where the EHDV-6 isolates were collected during a country-wide 2015 epizootic that affected dairy and beef farms and caused febrile illness with a variety of clinical signs including reduced milk production, lameness, weight loss, abortion, and some deaths [46]. A subsequent outbreak of EHDV-1 in cattle in Israel in 2016 was less extensive and milder with primarily subclinical infections; however, some milk-yield reduction, fever, and recumbency were observed [45]. These recent EHDV outbreaks in cattle are among several that underscore concerns raised about EHD as an emerging disease of cattle. In the U.S., a large multi-serotype outbreak across 15 states which caused clinical disease and deaths in cattle occurred in 2012 [14]. This was followed by a 2013 outbreak of smaller geographic scope, but with an unusually high cattle case fatality rate of 26% (16/61) of EHDV-infected animals [50]. A recent study comparing the viral genomes of EHDV-2 isolates taken from deer and cattle during the 2012 event in the U.S. did not identify significant genetic changes to support an increased virulence of these viruses, and suggested that environmental factors such as drought conditions likely contributed to the increased morbidity and mortality in cattle [51]. The 2013 U.S. outbreak reportedly was also preceded by drought in severely affected areas [50]; however, EHDV outbreaks do not appear to be universally associated with drought conditions as demonstrated in a 2017 WTD outbreak in the Eastern U.S. [52]. On a worldwide level, it is unclear whether the apparent increase of disease severity noted in EHDV-infected cattle is due to changes in climate, virus virulence, better detection methods, or any number of other inter-related, yet unknown factors. Regardless, continued diligence in surveillance and diagnostic testing is warranted to safeguard both livestock and wildlife health.

## 3. Target Species Yield Insights into Pathogenesis and Countermeasures

### 3.1. WTD In Vivo Studies

A well-defined animal model is a quintessential research tool for advancing knowledge of pathogen transmission and pathogenesis, and an absolute requirement for countermeasure discovery and development. Despite the inherent challenges of working with WTD in research settings, experimental infections in deer have provided insights into host susceptibility, pathogenesis, and immune responses since the initial determination that EHD was a viral disease [10,64,65,66,67,68,69,70,71,72,73]). Recent advances using in vivo WTD studies include the determination that deer were susceptible to experimental challenge with the emerging serotype EHDV-6, and that the EHDV-6-infected animals presented with clinical signs, pathological abnormalities, and postmortem findings consistent with studies of other EHDV serotypes in WTD [71]. In a recent experimental study, a WTD model was used to show that the infection rate of the midge vector *C. sonorensis* fed on EHDV-infected deer coincided with the level of viremia. The authors concluded that WTD are most infectious to midges during peak viremia which occurs shortly after EHDV infection [74]. Unexpectedly, virus was also isolated from midges that fed on animals with viremia below the limit of detection at later time points post-challenge, illustrating the invaluable role that in vivo studies play in investigating the poorly understood and highly complex interactions of vectors, viruses, and hosts in arbovirus transmission. The WTD challenge model has also recently been expanded to study the influence of maternal immunity in the EHDV susceptibility of young fawns. Previously, studies showed that maternally-derived neutralizing antibodies against EHDV were detectable in fawns until 17–18 weeks of age [75]. A recently developed WTD fawn challenge model addressed the question of whether such maternally-derived antibodies against EHDV are protective against challenge; in this study it was demonstrated that even low titers of maternal antibodies to EHDV-2 provided protection against clinical disease and greatly reduced levels of viremia following challenge with the homologous EHDV-2 serotype virus [72]. Finally, the WTD model was recently applied to vaccine discovery, enabling the evaluation of immunogenicity and efficacy induced by a novel subunit vaccine candidate comprised of the recombinant viral capsid VP2 protein of EHDV-2 [73]. This vaccine, which was efficacious in protecting WTD from viremia and clinical disease following an experimental challenge with a virulent EHDV-2 virus, has additional benefits including potential Differentiating Infected from Vaccinated Animals (DIVA) compatibility and wider safety margins than live-attenuated vaccines. Field trials of this vaccine, and others [76], are underway to determine their real-world impact on prevalence and severity of EHD in WTD populations in the field.

### 3.2. Cattle In Vivo Studies

An apparent recent increase in reported clinical disease among cattle has highlighted the need for additional studies of EHDV in domestic bovids. Some of the recent WTD studies which are discussed above also provided insights into cattle responses to EHDV. An EHDV-6 pathogenesis study in cattle found that viremia and seroconversion were more variable in cattle challenged with the same virus as WTD, and none of the cattle demonstrated clinical, hematologic, or pathologic abnormalities [71]. These findings were consistent with previous studies in which cattle experimentally infected with EHDV-6 developed no, or very mild, clinical signs [77,78]. Cattle vaccinated with the recombinant VP2 proteins of EHDV-2 and EHDV-6 produced virus-neutralizing antibodies against their homologous virus serotypes; the ability to neutralize heterologous viruses was limited [73]. A recent cattle challenge study, designed to generate a panel of bovine reference sera, provided data regarding the presence and absence of cross-neutralization between serotypes [79]. In seven calves, each challenged with one virus representing one of the seven EHDV serotypes (EHDV-1-2, and -4-8), viral RNA was detectable in all animals, and all seven seroconverted. No, or only weak cross-neutralizing reactivity was observed with most of the sera, with stronger cross-reactivity observed between some isolates of EHDV-2 and -7, and EHDV-6 and -8. Notably, the calf inoculated with the EHDV-7 developed some clinical signs leading to euthanasia, but it is unclear whether the clinical signs were due to EHDV because no postmortem examination was performed, and disease was not observed in prior cattle challenges with the same virus serotype [80]. Finally, in a cattle study investigating pregnancy and maternal immunity performed in Japan, where live-attenuated and inactivated EHDV vaccines are used to control natural infections of the virulent Ibaraki strain of EHDV-2, it was shown that the live-attenuated vaccine was safe for use in late term pregnant cows, and that calves did not seroconvert to EHDV following colostrum ingestion [81].

## 4. New Research Tools Aid the Development of Diagnostics and Countermeasures

### 4.1. In Vitro and Proxy Model Systems

Not all EHDV researchers have the resources and facilities to perform experiments in target animals, particularly in nontraditional research species such as WTD. Therefore, mammalian cell lines such as baby hamster kidney (BHK) cells, African green monkey kidney (Vero) cells, and calf pulmonary aortal endothelial (CPAE) cells are commonly used for in vitro studies of virus growth kinetics as well as for virus isolation and propagation [82]. The highly controlled environment afforded by in vitro cell culture systems can also facilitate the more mechanistic investigations of virus–host interactions. Recently, studies using bovine carotid artery endothelial (CAE) cells provided insights into the mechanisms by which EHDV-2 can induce cell death via apoptosis [83]. Studying EHDV transmission in vitro has provided unique challenges owing to the need to replicate the interactions between virus, mammalian host, and arthropod vectors. To address the need for additional laboratory-based research tools to study EHDV transmission by biting midges, the utility of the embryonated chicken egg (ECE) model, which has previously been used to study bluetongue virus (BTV), was demonstrated [84]. The ECE study showed that the three currently circulating North American EHDV serotypes (EHDV-1, -2, and EHDV-6) could be transmitted from an infected ECE to naïve midges, with subsequent virus transfer from infected *C. sonorensis* midges to naïve ECEs, the latter observed with EHDV-1 and -2 but not EHDV-6. In addition to identifying a method to investigate vector competence, the ECE study identified the potential need for further study of *C. sonorensis* competency for EHDV-6.

### 4.2. Molecular Vaccine and Diagnostic Tools

Recent advances in molecular biology will afford researchers the ability to investigate the influence of EHDV genetics on virus replication, pathogenesis, virulence, and immunity, as well as to develop rationally designed candidate vaccines. Virus-like particles (VLPs), which previously have been used to develop vaccines for, and study the biology of, BTV, have been generated using recombinant structural proteins of EHDV. Four viral capsid proteins (VP2, VP3, VP5 and VP7) of a field isolate of EHDV-6 were shown to assemble into VLPs with correct morphology and size [85], thus providing new options for the expression of modified viral proteins. In another study, the potential for VLPs to act as effective immunogens was explored using VP2, VP3, VP5 and VP7 of EHDV-1. These capsid proteins were expressed simultaneously using a baculovirus expression vector expressing multiple antigens. The generated VLPs produced strong neutralizing titers in immunized rabbits, demonstrating that the EHDV capsid proteins were presented to the rabbits’ immune system in the correct immunogenic conformations [86]. A plasmid DNA-based reverse genetics platform was also recently described, which could aid researchers in generating modified live viruses for vaccine development or studies on virus pathogenesis and virulence [87]. Targeted and rational design of EHDVs using the reverse genetics system may be further aided by methods such as structural modeling and epitope prediction. In a recent comparison of orbivirus sequences, consensus sequences for the VP5 and VP7 proteins of EHDV, BTV, and AHSV were obtained, and homology models were constructed to fill in knowledge gaps regarding the structure of EHDV proteins [88]. Subsequent computational analyses identified and mapped potential linear and discontinuous epitopes in these structural proteins of the inner and outer capsid proteins.

New molecular tools have also been developed to help diagnose EHDV more efficiently, and with increased sensitivity and specificity. TaqMan real-time RT-PCR (RT-qPCR) assays were recently described for both pan-reactive and serotype-specific detection of EHDV-specific RNA, with the ability to detect as few as two copies of viral RNA per reaction [89]. A method for the molecular detection of EHDV infection postmortem was described using bone marrow collected from WTD, providing new options for virus surveillance. Following sequential harvesting of bone marrow from deer carcasses with previously confirmed EHDV infections, EHDV-specific RNA could be detected by RT-qPCR in bone marrow for up to 12 weeks after death [90]. Other advances in diagnostics include new options for serological screening to detect evidence of EHDV infections. A competitive ELISA (c-ELISA) using unpurified baculovirus-expressed VP7 was described as an option for researchers limited by time or resources to perform antigen purification [91]. A new commercial VP7 c-ELISA test was evaluated to address a critical gap left by a recently discontinued commercial testing kit. The new VP7 c-ELISA test was observed to have limited cross-reaction with BTV-positive cattle sera, and a comparable sensitivity to the discontinued diagnostic product [92]. A c-ELISA to detect BTV IgM antibodies was also tested for cross-reactivity with EHDV, and was found to detect EHDV-6 IgM antibodies in a portion of sera from cattle experimentally infected with EHDV-6 [93]; this suggests that there are diagnostic tools with the potential to detect antibodies specific for the acute phase of EHDV infections. Most recently, a fluorescent microsphere immunoassay (FMIA) using whole-virus antigen preparations was developed with the capability of differentiating bovine antibodies to BTV and EHDV in a single, small volume serum sample [94].

## 5. Developments in Vector Control

In the absence of safe and efficacious vaccines, biting midge vector management remains the best available method to reduce EHDV transmission for wild cervids and domesticated animal populations such as pastured beef and dairy cattle, sheep, or captive cervids. Insect vector management (IVM) reduces biting midge populations and limits contact (feeding) events between vector insects and host animals; this will decrease virus transmission during outbreaks if the vector species are known [52,95]. Furthermore, by reducing adult midge survival, the age distribution shifts towards younger midges which are less likely to be infected and infectious when they bite host animals [96]. Current IVM methods (reviewed in [22,97,98]) use larval habitat reduction and chemical treatments to target immature larval stages while they are confined to semi-aquatic habitats such as moist substrates or feces. Aerial spraying of pesticides targets the more mobile adult stages. However, for free roaming susceptible animal populations, insecticides applied to larval habitats or adult resting sites are nearly impossible to apply efficiently due to the extensive effort and amount of product required to treat a large geographic area effectively. Animal treatments such as long-lasting controlled release of insecticides from ear tags, pour-ons, or systemic insecticidal feeds are not very effective because the biting midges are only in contact with the animals for a short period of time and can pick up the virus even from animals with low-titer viremia [74]. Treating animals or the environment for insect management must be done properly and within label recommendations or the insecticides may eventually have detrimental impacts on the environment and the animals. Insecticide (e.g., imidacloprid) contamination has been found in high concentrations in wild deer populations [99] with adverse impacts such as developmental abnormalities observed in fawns; however, it must be stated that imidacloprid is not typically a biting midge pesticide, and more typically used as a seed treatment for crops which wild animals may be exposed to via bioaccumulation.

To reduce pesticide exposure and non-target impact, new insect-specific management methods are being developed as next-generation pesticides; this technology is based on successes in mosquito management [100]. Sterile insect technique using *Wolbachia* infections may be possible after finding natural populations of midges infected with *Wolbachia* [101], and also with the first successful infection of cell lines derived from the vector *C. sonorensis* with exotic *Wolbachia* [102]. Additional recent advancements with RNAi technology [103,104] may provide a foundation for the development of species-specific treatments, although these next-generation tools are far from being available. A significant problem with species-specific population management is that new vector species continue to be identified each year [105,106,107], and the new tools are usually highly species-specific. Continued characterization of outbreaks, the environmental drivers, and insect vector compositions involved in new EHDV outbreaks are critical to understand the changing epidemiology [52,95,108]. Additional research hurdles still exist; field trials must be performed and deployment issues such as regulatory and end user acceptance must be overcome (reviewed in [100], but the arrival of new control tools to reduce biting midge populations which are more environmentally sustainable is expected in the near future.

## 6. Outlook

The advent of safe, effective, and fully licensed, commercially available vaccines for EHDV will provide the potential to significantly change the landscape of EHDV epidemiology and the damaging impacts that EHD historically has had on farmed, and other captive, North American WTD populations. The financial effects of EHD on the deer farming industry are difficult to determine, in part because the disease is clinically indistinguishable from a similar disease caused by BTV infection; however, reports by farmers of devastating animal losses are common during EHDV outbreaks, with mortality rates of up to 80% [109]. Ideal strategies for vaccine deployment will depend on the duration of immunity observed in vaccine field studies of deer that are ongoing in the U.S. Other factors that will need to be considered include seasonal timing to ensure that animals have sufficient protection during periods of peak insect activity and transmission, existing herd health management and handling schedules on deer farms, and balancing potential interference of maternal immunity with vaccinating fawns too early after birth in the presence of passive antibody protection. Meanwhile, the changing global climate may result in expansion of vector species to new areas that were previously vector-free. Warming temperatures may also result in altered vector competence of some midge species, also increasing the risk of EHDV outbreaks in new areas. These issues emphasize the need to combine vaccinations with integrated vector management plans when moving forward.

Ideally, EHDV vaccination would protect animals against the different EHDV serotypes that are present in a given region. However, the ability of the vaccines under development to generate durable universal immunity that can cross-protect between different EHDV serotypes remains uncertain. Observations from the cattle study mentioned above suggested that there is no or only limited cross-neutralization between EHDV serotypes 1, 2 and 6, but stronger cross-reactivity between EHDV-2 and -7, and EHDV-6 and -8 [79]. This is in contrast with a 2002 study in WTD which observed that fawns previously infected with EHDV-2 experienced reduced clinical disease when subsequently challenged with EHDV-1. However, high neutralizing antibody titers to EHDV-2 were not associated with decreased clinical disease severity following EHDV-1 challenge, suggesting that cell-mediated immunity may play a larger role in cross-protection than previously thought [110]. Regardless, it may ultimately be determined that a multivalent EHDV vaccine, or multiple monovalent vaccines, may be required to ensure sufficient protection for at-risk herds in certain endemic regions. A multivalent vaccine approach would increase development costs and costs to producers but may ultimately be cost-effective when weighed against potential herd losses. Implementation of effective EHDV vaccines could also have additional secondary benefits. EHDV-infected WTD often develop a reduction in peripheral lymphocytes (i.e., lymphopenia) during the acute phase of infection and are, therefore, considered vulnerable to secondary infections [12,68]. Consequently, a vaccine-based protection from EHDV-mediated lymphopenia could have a potential benefit of reducing opportunistic secondary microbial invasion in EHDV-infected WTD and reduce the morbidity and/or mortality from other infectious causes. Lymphopenia has not been described as a feature of EHD in cattle; however, the experimental reproduction of the disease in cattle has been rather challenging [111]. Infection-related fatalities in cattle have generally been attributed to sequelae rather than acute EHD [50]. This highlights the critical need to better understand EHDV pathogenesis in cattle in order to evaluate and mitigate potential impacts of EHDV to the economically important cattle industry. Another potential benefit of vaccination with an effective subunit vaccine, which is not offered by a modified live virus (MLV) vaccine, may be the reduction of the amount of virus in the environment. Because sterilizing immunity has been demonstrated by at least one vaccine in development [73], these vaccine formulations may have the ability to reduce the transmission of virus from mammalian hosts to its insect vectors.

The emerging patterns exhibited by the ever-evolving epidemiology of EHDV in recent years make the prospect of virus control somewhat daunting. The expanding geographic distribution of various EHDV serotypes and their vectors, and the increasing severity and frequency of outbreaks in both deer and cattle, provide new challenges. However, the growing body of countermeasure tools, resources, and information available to scientists, diagnosticians, clinicians, and producers is poised to yield new, more effective approaches to meet these challenges in the near future. There are ample reasons to believe that the EHDV control landscape, while formidable, looks increasingly promising.

## Figures and Tables

**Table 1:** Notable detection of EHDV serotypes.

Serotype	Year	Country	Source	Reference(s)
Type 1	1955	USA	Deer	[9]
Type 1 (originally 3)	1967	Nigeria	*Culicoides*	[53]
Type 1	1984	Japan	Cattle	[48,54]
Type 1	1992	Australia	Cattle	[55]
Type 1	2015	Ecuador	Cattle	[43]
Type 1	2016	Israel	Cattle	[45]
Type 1	2016-2017	Egypt	Cattle	[38]
Type 2 (Ibaraki)	1959	Japan	Cattle	[31]
Type 2	1962	Canada	Deer	[26,27]
Type 2	1979	Australia	Cattle	[56]
Type 2	1996	USA	Cattle	[6]
Type 4	1968	Nigeria	*Culicoides*	[53]
Type 4	1982	Sudan	*Culicoides*	[57]
Type 5	1977	Australia	Cattle	[56]
Type 5	1991	Indonesia	Buffaloes, cattle, & sheep	[58]
Type 6	1981	Australia	Cattle	[56]
Type 6	2006	USA	Deer, cattle	[28]
Type 6	2006	Morocco	Cattle	[59]
Type 6	2007	Turkey	Cattle	[4]
Type 6	2013	Trinidad, West Indies	Deer, cattle	[39]
Type 6	2016	Mayotte	Cattle	[40]
Type 6	2021	Libya	Sheep	[60]
Type 7	1981	Australia	Cattle	[56]
Type 7	1997	Japan	Cattle	[8,48]
Type 7	2006	Israel	Cattle	[5,61]
Type 7	2018	China	Cattle	[49]
Type 8	1982	Australia	Cattle	[56]

Not exhaustive; for additional information, see also references [33], [62], and [63].
